# Colour Preference of Post Hatchling Hawksbill (*Eretmochelys imbricata*) and Green (*Chelonia mydas*) Sea Turtles in Captivity

**DOI:** 10.3390/ani15050628

**Published:** 2025-02-21

**Authors:** Jordan Drake, Mohammed F. Khayat, Rhondda Jones, Ellen Ariel

**Affiliations:** 1 College of Public Health, Medical and Veterinary Sciences, James Cook University, 1 Solander Drive, Douglas, QLD 4811, Australia; mfakhayat@kau.edu.sa (M.F.K.); rhondda.jones@jcu.edu.au (R.J.); ellen.ariel@jcu.edu.au (E.A.); 2 Marine Biology Department, Faculty of Marine Sciences, King Abdulaziz University, Jeddah 21589, Saudi Arabia

**Keywords:** hawksbill turtle, *Eretmochelys imbricata*, green turtle, *Chelonia mydas*, colour vision, behavioural responses, ecological niche

## Abstract

Variations in the ecological roles of sea turtle species may lead to differentiations in ocular design and visual sensitivity to the colour spectrum. Behavioural colour preference studies in air and in water on hatchling and post-hatchling green turtles found evidence of a blue hue attractiveness when given a choice between blue, red, and yellow. This paper assessed and compared the colour preferences to singular colours via the behavioural responses of eleven hawksbill turtles and twelve green turtles at 15 months of age and at 22 months of age. Turtles were presented with one coloured water balloon per day (purple (400–450 nm), dark blue (450–490 nm), cyan (490–520 nm), green (520–560 nm), yellow (560–590 nm), orange (590–635 nm), and red (635–700 nm)). Time to contact balloons with beak and behaviours exhibited by turtles were recorded. Hawksbill turtles had the greatest level of interactions across both phases to shorter wavelengths with hue preference being between 450 and 490 nm. Green turtles consistently had the greatest level of interaction to longer wavelengths with a yellow (560–590 nm) hue preference. The results of this study support behavioural differences between two co-occurring turtle species that may reflect an adaptive preference for colour wavelengths associated with the optimal foraging niche for each.

## Simple Summary

Sea turtle vision has adapted to life in air and in water up to great depths. In some studies, the visual capability of certain turtle species to see and respond to colour have found turtles can see most of the wavelengths within the visible and ultraviolet spectrum. Using coloured water balloons, we compared the behavioural responses and colour preferences of hawksbill and green turtles as they aged from 15 months to 22 months. We found that hawksbill and green turtles reacted differently to each of the colours tested. Hawksbill turtles had an attraction to shorter wavelengths such as dark blue and cyan, and green turtles had an attraction to longer wavelengths with a consistent yellow hue preference. The differences found between these species likely result from the different habitats and depths that they occupy and the effect of depth on longer wavelengths.

## 1. Introduction

Receiving and responding to environmental feedback is crucial for organism survival [1] as the sensory system connects biological, ecological, and physiological functions to that of the ever-changing environment. Perhaps the most important sense for many animals is vision, which comes in varying forms of complexity in animals of many phyla, suggesting a generalised necessity for spectral information [2]. The capabilities of a sensory system, colour vision in the visual system for example, is closely linked to how it serves the animal behaviourally [2] and in how the animal perceives it rather than the inherent property of an object [3].

Colour vision is useful for recognising objects and assigning value to them such as courtship displays [4], warning coloration [5], crypsis and mimicry [6], foraging selection [4], and sheltering [1]. Conspecific communication through use of colour cues has been well documented in dichromatic species of birds [7,8,9], lizards [10], and in fish [11,12] during courtship displays and mate selection. Conspicuous coloration and flashy showmanship from males are often actively selected for by females, and bright coloration is argued to be directly correlated with high-quality individuals [10]. Chromatic signals are not only used in communication with conspecifics but also in animals’ response to and interaction with the surrounding environment, including during foraging. Plants that utilise animals for pollination and seed dispersal have co-evolved colour signals with the spectral absorption of the recipients to communicate fruit ripeness and seasonal pollinator cues [13,14,15,16].

The perception of a light reflected off an object will differ depending on the animal’s absorption spectra photoreceptors [3], leading to different colour vision capabilities across the animal kingdom. To determine the spectral sensitivities of colour vision in animals, behavioural colour discrimination experiments are often used to determine which colours animals can see by training animals to select one colour against another [3,17], discrimination of the same colour at different intensities, and discrimination of one colour from a series of grey shades [14]. In behavioural colour discrimination experiments, a level of learning or training is required, while colour preference involves the selection of colours without any training or reward and could indicate innate ecological needs associated with specific wavelengths [2].

Not all animals are accessible participants to behavioural studies, be that they are endangered or hard to find in nature, so other physiological methods like electroretinogram and microspectrophotometry from DNA sample or post-mortem testing can conclude that animals have the capacity to differentiate different light wavelengths [18]. However, animals with three or more photopigments, which would indicate the capability to differentiate wavelengths and see colour, have been found through behavioural studies to not use all the photoreceptors to discriminate certain wavelength ranges [3,19]. Therefore, it is important to remember that even animals that have the physiological ability to differentiate wavelengths may not react to these wavelengths in behavioural trials, indicating that there are behavioural adaptations to certain wavelengths in their environment. Ideally, evidence for colour vision should stem from a combination of physiological and behavioural studies when possible.

The adaptive benefits of the visual system, and colour vision, vary across species and life stages, allowing opportunities for animals to develop environmental niches and adapt to different lifestyles [13]. When dealing with life-stage migratory species such as sea turtles, it is therefore especially important to carry out trials on these threatened species at different age classes even if it is on a small scale to gain a better understanding of how these animals interact with their environment through use of colour vision.

There is evidence of colour vision in sea turtles from a handful of studies focusing on green and loggerhead (*Caretta caretta*) turtles. Several studies have found a blue hue preference in hatchling green turtles (*Chelonia mydas*) under various stimuli and frequency intensities in air and water studies [20,21,22,23]. Adult loggerhead turtle studies have found similar evidence for blue hue attraction when given the choice between colours [20]. It is thought that this preference for blue is an innate attraction due to the sea-finding behaviour of hatchlings [22,24], and it helps to distinguish food from the blue background of their marine environment [18,25,26]. When presented with a choice between blue and another colour, as seen in previous studies, turtles may be selecting blue over the other colour due to the function blue serves in their environment. Each turtle species and age class occupy distinct ecological niches, such as the deep-water dwelling leatherback turtle (*Dermochelys coriacea*) for which visual requirements and capabilities would likely differ from those of shallow-water green turtles.

There have been limited studies comparing the visual capabilities of other sea turtle species outside of green and loggerhead turtles. The spectral sensitivities of hawksbill turtles (*Eretmochelys imbricata*) have not been measured in a behavioural or physiological sense, and their diet and habitat preferences differ from those of green and loggerhead turtles. This study aims to examine and compare the in-water hue preferences of hawksbill and green post-hatchling turtles through behavioural changes and variations in latency times.

## 2. Materials and Methods

### 2.1. Animals and Husbandry

This study involved 11 hawksbill and 12 green post-hatchling turtles, which were approximately 15 months old at the start of the study. All turtles were collected at emergence from nest on Milman Islet, Northern Great Barrier Reef, Australia, in March 2019. Hatchlings were collected under permits from the Department of Environment and Science (Ptu18-001419-2) and James Cook University Animal Ethics (A2586).

Post-hatchlings were housed at the Turtle Health Research Facility, ‘The Caraplace’, at James Cook University, Queensland, Australia. During phase A, turtles were housed in 1000 L rectangular pens divided into three compartments and during phase B, for hawksbill turtles only, they were divided into two compartments to accommodate their increased size. The depth of water within the pens was roughly 40 cm ± 5 cm. Each enclosure had a small stationary mesh platform to provide turtles a shelter and place to rest. Water quality was maintained via UV sterilisation, mechanical filtration through 50 µ filters, fractionation, and regular water exchange. Water temperatures were maintained at 27 °C ± 1 °C and salinity at 35 ppt ± 3 ppt.

During phase A, two pens with six green turtles and two pens with six hawksbill turtles were shaded with fine mesh shade sails, while the other four pens housing six green turtles and five hawksbill turtles were exposed to direct sunlight each day. During phase B, all pens were shaded with fine mesh shade sails.

During experimentation periods, turtles were fed at a rate of 5–7% their body mass per weekday. The food consisted of gelatine cubes containing blended human grade tinned sardines, vegetables, fish pellets, and Sea Tabs^®^ Antioxidant Vitamins.

### 2.2. Experimental Design

Phase A occurred during the winter season with clear, sunny skies, and phase B occurred during the summer season where days were dry but overcast. Phase A of the study was conducted when turtles were 15 months old, and phase B was conducted when turtles were 22 months old. Turtles were exposed to one of seven different coloured balloons per day for each phase and approximately 2.5 h after daily feeding. To control for light intensity changes throughout the day, experiments were conducted at approximately the same time each day. Turtles trialled in shaded pens were conducted from 11:30 a.m. to 12:30 p.m., and turtles in pens without shade sails were trialled between 12:30 p.m. and 1:30 p.m. During phase B, all pens were shaded, but the experimental time period was kept the same for each group of turtles.

Spherical balloons were employed during phase A, and cylindrical balloons were employed in phase B. All balloons were filled with water to a weight of 250 g. Balloons were tied off and attached to a 25 g sinker with clear fishing line. The sinker fixed the balloon in place and held it roughly mid-water, which was about 20 cm deep. The different balloon shapes for each phase provided a new shape to encourage a reaction to colours rather than shape and to overcome the influence of familiarity from the previous phase of the study.

Eight colours were selected for experimentation: dark blue (450–490 nm), yellow (560–590 nm), green (520–560 nm), orange (590–635 nm), cyan (490–520 nm), purple (400–450 nm), red (635–700 nm), and white (390–700 nm). White balloons were shown to six hawksbill and six green turtles prior to the start of the experiment to determine which behaviours were exhibited for the ethogram, as detailed below, but were not used in the experimental trials. The colours were presented in the order listed above as alternating short and long wavelengths.

#### 2.2.1. Set-Up

A GoPro Hero 4^®^ was mounted to a camera stand which could be positioned over each compartment. The GoPro was connected via Bluetooth to the GoPro App so recording could be controlled without being near the turtle. Remaining out of sight of the turtle limited the possible human influence on its behaviour.

Turtles were removed and placed into dry, covered buckets while the experiment was set up and while other turtles in the same pen were trialled to limit visual distractions during trials. Placing turtles in dry buckets provided a cool, shaded location with limited view which assisted in keeping them calm during the experimental setup and was part of daily husbandry. Platforms in each enclosure were removed and recirculation was turned off. The balloon and attached sinker were positioned where the platform had previously been in each enclosure and was placed in the same spot throughout all the trials.

#### 2.2.2. Recording Behaviour

Prior to the turtle being introduced to the balloon, video recording was started. One turtle at a time was reintroduced to their enclosure at the opposite end to the balloon. When a turtle was being transferred from the dry bucket to the enclosure, it was held until calm, which was defined as front flippers hanging down and limited head movement. If a turtle was waving its flippers and moving the head from side to side, it was placed back in its dry bucket for several minutes until it calmed down. The turtle was placed at 90° to the balloon to prevent gliding through the water toward the balloon simply as a result of release. This position allowed the turtles to see the balloon as soon as they contacted the water due to their horizontal streak, providing them with a panoramic field of vision [4].

Latency time, defined as beak to balloon contact, was recorded in seconds. A three-minute (180 s) time limit was established, and turtles that did not make contact within the time limit were recorded as 181. During the white balloon control trials, general behaviours were determined and defined including resting, diving, swimming along the enclosure wall or into the enclosure corner, splashing, flipper to balloon touch, carapace to balloon touch, and carapace turns (Table 1). These behaviours were recorded during trials and used for supplementary data when looking for species behavioural trends as well as looking for signs of agitation in individuals. Trials were ended based on three situations: behavioural signs of agitation that persisted beyond half the trial time, upon contact with beak to balloon before 180 s, and if no contact to the balloon was made within 180 s.

### 2.3. Data Analysis and Statistics

Statistical analysis used R version 4.2.1 and RStudio 2022.07.1. Statistical significance used a critical *p*-value of 0.05.

#### 2.3.1. Analysis of the Probability of Contact with a Balloon

Due to the small sample size and the need to test for interactions among explanatory variables, there were not enough data to support analyses incorporating 6 different balloon colours, so colours were grouped into short wavelengths (purple, blue, and green) and long wavelengths (yellow and orange). Red balloons were not contacted by any turtles in either phase A or phase B and were therefore excluded from all analyses.

For phase A, factors affecting the proportion of turtles contacting balloons with their beak within the 180 s trial limit were assessed using a mixed-effects logistic regression with balloon colour, species, and their interaction as fixed effects and individual turtle as a random effect. This mixed-effects model was compared to logistic regression without random effects to evaluate evidence for differences between individual turtles. Analysis of deviance (type 2) was used to determine the statistical significance of each fixed effect.

When phase B data became available, the logistic regression executed for phase A was repeated using all available data and including phase and interactions involving it as fixed effects.

#### 2.3.2. Analysis of the Time Taken for Turtles to Contact Balloons

The effects of short vs. long wavelength balloon colours and turtle species on the time taken for turtles to contact balloons were evaluated using Cox regression and illustrated using Kaplan–Meier curves. The same approach was used to examine whether the time to balloon contact during phase A was influenced by whether trials were carried out under mesh shade sails or in direct sunlight.

#### 2.3.3. Mass and Contact Time, and Light Intensity Comparison

Cox regression was used to test if body mass, balloon colour, species or phase influenced the time to contact a balloon.

## 3. Results

Eleven hawksbill and twelve green turtles went through two experimental phases each, with each turtle exposed to the seven balloon colours once during each phase, for a total of 14 trials for each turtle. Including individual turtle ID as a random effect did not improve the explanatory power of logistic regression analyses (LR X^2^ = 0.487, 1 df, *p* = 0.485, higher AIC for mixed effects model). This indicates that individual turtles did not differ significantly in their responsiveness to the balloons.

### 3.1. Probability of Contact with a Balloon

**Figure 1:**
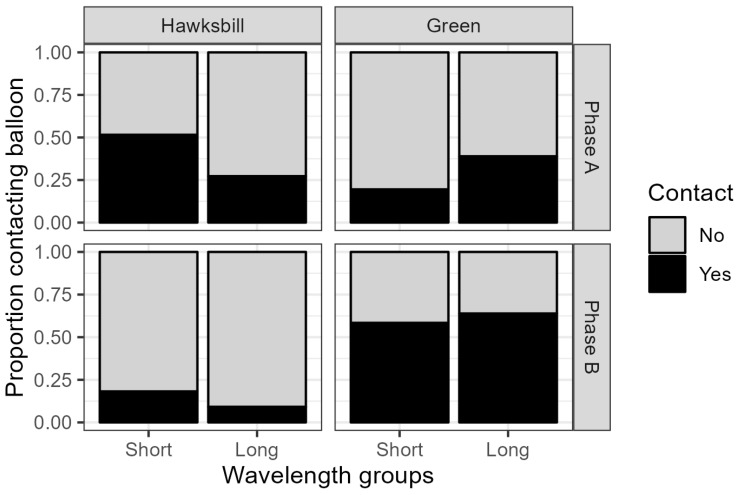
The effects of turtle species, wavelength group (balloon colour), and phase on the estimated probability of contact with a balloon. Colours were grouped into short (purple, dark blue, light blue, and green) and long (yellow, and orange) wavelengths, omitting red from the groupings as no turtles ever contacted red balloons.

In an initial analysis examining only phase A, there was no difference between species in the overall proportion of turtles contacting a balloon. However, the pattern of colour preference differed markedly between the two species, as shown by a significant interaction between balloon colour and species (logistic regression ANOVA: df = 6, LR X^2^ = 29.1018, *p* = 5.820 × 10^−5^): that is; hawksbill turtles were more likely to contact balloons with a short wavelength colour, whereas green turtles were more likely to contact balloons with a long wavelength colour (yellow or orange) (Figure 1).

Subsequent analysis applied a logistic regression to data from both phases, examining the effects of species, short vs. long wavelength colours, and phase (age when tested), as well as all possible interactions between these variables, on the probability of contact with a balloon (Figure 1). There were two significant outcomes of this analysis. First, there was a species–wavelength interaction. As in the initial analysis of phase A data, hawksbill turtles were more likely to contact balloons with a short wavelength colour, whereas green turtles were more likely to contact balloons with a long wavelength colour (LR X^2^ = 8.11, df = 1, *p* = 0.0044). Secondly, there was a species–phase interaction. In phase B, green turtles were just as likely to contact balloons as they were in phase A, but hawksbill turtles were much less likely to contact balloons than they had been during phase A (LR X^2^ = 27.25, df = 1, *p* < 0.0001).

No significant three-way interaction of phase, wavelength group, and species on colour preference was found (LR X^2^ = 0.6878, df = 1, *p* = 0.401). Although colour preference appeared weaker in phase B (Figure 1), its direction remained similar in both phases; specifically, hawksbill turtles tended to prefer short wavelengths and greens tended to prefer long wavelengths.

### 3.2. Time Taken for Turtles to Contact a Balloon

The speed of contact for hawksbill turtles was significantly faster at short wavelengths than at long wavelengths (Kaplan–Maier: df = 1, chisq = 10.6, *p* = 0.001), but the effect of wavelength was not significant for green turtles’ speed of contact (Kaplan–Maier: df = 1, chisq = 1, *p* = 0.3.

**Figure 2:**
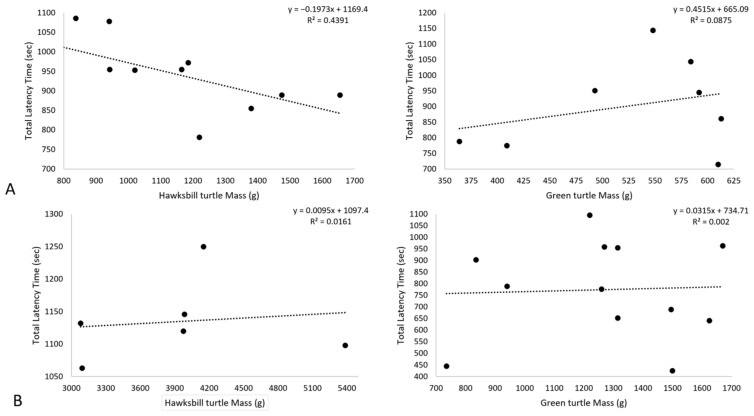
Total latency time in seconds (s) of beak-to-balloon contact and individual mass (g) of hawksbill turtles (*n* = 11) and green turtles (*n* = 12) for phase (**A**) (top row) and phase (**B**) (bottom row).

During phase A, the hawksbill turtle mass averaged 1013.95 g (515.6 to 1480 g). A significant correlation of individual mass and mean contact time showed that larger hawksbill turtle individuals had shorter mean contact times across the coloured balloons (linear regression df = 11, r^2^ = 0.4391, x^2^ = −0.663) (Figure 2A). Hawksbill turtles during phase B averaged a mass of 3949.33 (5385 to 2870 g). There was no significant effect of mass on total latency time for hawksbill turtle individuals during phase B (linear regression df = 10, r^2^ = 0.0063, x^2^ = −0.1171) (Figure 2B).

During phase A, the green turtle mass averaged 460.35 g (303.20 to 596.80 g). Mass had no significant effect on the mean contact time of green turtle individuals (linear regression df = 8, r^2^ = 0.0875, x^2^ = 0.3) (Figure 2A). During phase B, green turtles’ average mass was 1265 g (735 to 1670 g). No significant effect of mass on total latency time was seen for green turtles in phase B (linear regression df = 11, r^2^ = 0.002, x^2^ = −0.11) (Figure 2B).

**Figure 3:**
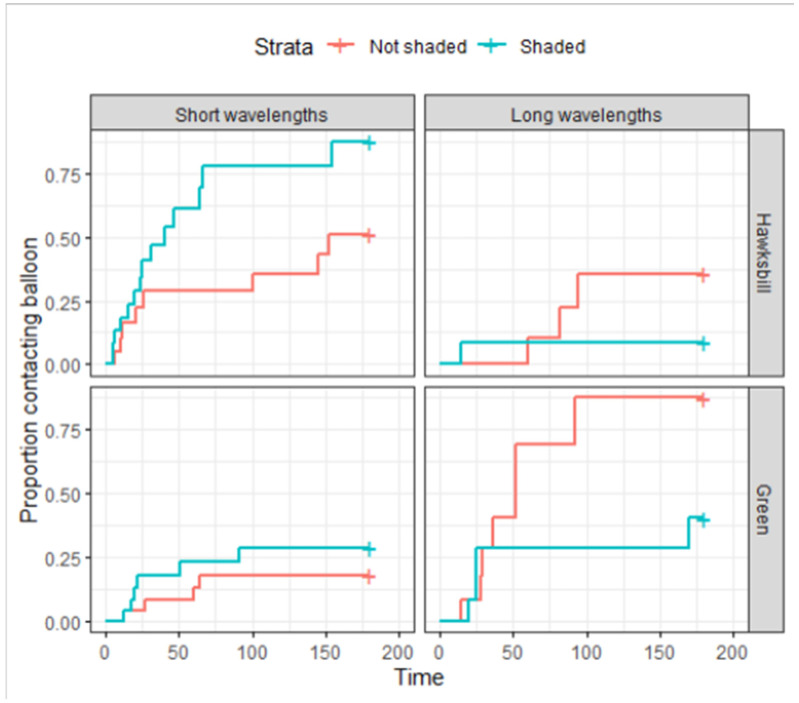
Times to balloon contact, in seconds (s), of post-hatchling hawksbill turtles and green turtles in which six hawksbill turtles and six green turtles were shaded with fine mesh shade sails throughout trials (blue), and five hawksbill turtles and six green turtles were exposed to direct sunlight during trials (red). Shading appears to slow contact times for long wavelength colours and to increase it for short wavelength colours.

In phase A, a group of six hawksbill turtles and six green turtles were under fine mesh shade sails throughout the data collection, while the remaining six green and five hawksbill turtles were exposed to direct sunlight. During phase B, all turtles were under shade sails throughout data collection. The shade sails did influence the time to contact wavelengths, increasing time to contact short wavelengths and decreasing time to contact long wavelengths (LR X^2^ = 4.2067, df = 1, *p* = 0.040265) (Figure 3). Interestingly, hawksbill turtles respond differently to coloured balloons in the shade than in direct sunlight, while green turtles responded differently to coloured balloons in direct sunlight than in the shade (LR X^2^ = 10.4839, df = 1, *p* = 0.001204) (Figure 3).

## 4. Discussion

Behavioural responses to submerged objects in the colour range of 400–700 nm revealed a difference between young hawksbill and green turtles, which may reflect a difference in their ecological needs to differentiate across wavelengths.

The great advantage of the cohort of turtles examined here is that they have a known and similar history of exposure in their environment prior to the trials. This allowed us to test their initial/innate reaction to coloured items. Logistics and permit requirements did put a limit on the number of animals in trials. This was compensated for in the analysis by the grouping of colours into short and long wavelengths. There may be individual differences in response to colour within each wavelength group, which would require a much larger sample size to identify; however, working with wild and threatened species requires specific permits which often comes with a limit on numbers based on population size rather than on many replicates in an experiment [27,28].

The defining factor of colour vision is not only that the animal has the appropriate visual pigments within cone cells, as that is only evidence of ability to register a particular light wavelength, but that the animal can perceive and respond behaviourally to the signals received [18]. This assumes that animals are tested in the colour range that they are capable of seeing. Oil droplets within the cone cells of the retina create a wider range of wavelength absorption by photoreceptors where the type and combination of droplets determines the range of colour visible to the animal. Green turtles have three types of oil droplets, clear, yellow, and orange, which stretches the spectral sensitivity from the ultraviolet spectrum (10 to 400 nm) to a peak absorption of 600 nm in the visible spectrum [18,29,30,31]. The colour red has a wavelength range of 635–700 nm, which is longer than the peak absorption range of green turtles, suggesting green turtles cannot see the colour red. This has been further supported by this study in phase A and B where none of the green turtles contacted the red balloon. The wavelength absorption range has not been identified in hawksbill turtles, but similar peak sensitivities have been found through the electroretinography of hatchling loggerhead turtles [30], hatchling leatherback turtles [30], and adult loggerhead turtles [29]. This study found none of the hawksbill turtles contacted the red balloon in either phase, suggesting a similar peak sensitivity to green, leatherback, and loggerhead turtles. Interestingly, zero hawksbill turtles contacted the orange-coloured balloon (590–635 nm) in either phase, suggesting a difference in the absorption properties of the photoreceptors. Electroretinogram and microspectrophotometry analyses of hawksbill turtles would further provide evidence of their spectral sensitivities to longer wavelengths.

Most colour vision studies on sea turtles present colours using projected lights in the varying wavelengths and intensities, so when a turtle avoids or selects against red, studies suggest the light is simply invisible to them [32]. In the case of this study, a physical object coloured red was in the tank, so if turtles were behaving normally as a result of the object being invisible to them, some accidental touching of the balloon should have been observed. For every trial, behaviour notes were taken using the ethogram to identify signs of stress in turtles as a response to any colours (Table 1). For the red-coloured balloon trials, in both phase A and phase B, green and hawksbill turtles spent the trial time swimming laps around the experimental tank, looking at the balloon up close before returning to swimming laps. Few turtles remained at the opposite end of the tank from the balloon, indicating a drastically reduced response to the red-coloured balloons. A similar behaviour to red light at high intensities was seen in loggerhead turtles and was revealed as confusion rather than avoidance [23,32]. It is possible the light intensity of the red-coloured balloons was just high enough for the green and hawksbill turtles of this study to register some sensitivity to the long wavelength but perceived as barely visible to the animal. Light intensity of the coloured balloons was not tested or controlled for, so it is unclear why the turtles in this study showed some level of confusion or mild avoidance to the red-coloured balloons and why no accidental contact was observed.

In the wild, turtle behaviour toward coloured objects may be influenced by other environmental factors. Environmental conditions influence visual capabilities, and the significance of that effect is stronger during developmental stages [20,33]. The expression of short, medium, or long cones within the retina could be compromised due to environmental conditions such as light intensity or exposure to colour [33]. The turtles in this study had never lived in the ocean prior to the start of the experiment, and exposure to colour was limited to the blue feeding bins used during husbandry, the grey housing troughs, and clothing from the volunteers and researchers. The findings reported here were obtained under the set and described conditions where the only variable was the change in colour of the balloons. Colour vision and thus the spectral sensitivity of photoreceptors is largely influenced by light intensity [34]. In air, light intensity can be affected by environmental factors such as seasonal day–night lengths [35], weather such as cloud cover, and habitat structures such as shaded forests and exposed deserts [36,37]. In aquatic environments, light intensity is largely affected by depth [35], where longer wavelengths are absorbed in shallow environments, while shorter wavelengths are scattered and penetrate deeper [38,39,40], but also by other factors such as wind, currents, tide, waves, cloud-cover and suspended particles in the water. In the wild, pelagic stage turtles likely spend most of their time at the ocean surface and should be exposed to a broad spectrum of light wavelengths. During pelagic phases, turtle species are opportunistic omnivores, feeding on mixtures of algae, and predominately zooplankton found in surface waters [41]. As adults, green and hawksbill turtles show adaptive preferences in foraging sites, diet composition, and habitat use that would reflect wavelength preference differences regardless of similar spectral sensitivities during their pelagic phases.

Adult hawksbill turtles forage on a variety of benthic organisms, primarily feeding on sponges. Studies have found similarities in sponge species consumed by hawksbill turtle populations in the Atlantic, Pacific, and Western Indian Oceans [42]. Trends have been found in the Caribbean to suggest hawksbill turtles may select sponges based on a criterion of abundance, nutrient levels, and chemical deterrents when given a variety of sponges to prey upon [43]. Hawksbill turtles diving and foraging on coral reefs may not utilise the ability to differentiate longer wavelengths, when shorter wavelengths would be more advantageous to their lifestyle. The hawksbill turtles of this study were most attracted to shorter wavelengths at both 15 and 22 months of age, which could suggest a shorter wavelength preference reflected even at a developmental age.

As adults, green turtles migrate from pelagic to nearshore neritic habitats and forage in shallow seagrass meadows. Living almost extensively within surface waters would suggest green turtles are exposed more frequently to longer wavelengths than species occupying deeper water, and they would therefore utilise the ability to differentiate across a larger spectrum of wavelengths. In this study, green turtles were most attracted to yellow-coloured objects across both phase A and phase B. Other in-water studies report blue as the most attractive colour when green turtles were presented with a choice between food on a blue, red, and yellow background [9]. It has been suggested that turtles prefer blue for the contrast of the prey or food item and the ocean background, but in shallow waters, turtles could select for the brightest photic stimuli, which would be the green–yellow wavelength range (520–590 nm) [44]. Selecting for the brightest colour would assist green turtles in foraging and selecting seagrass areas.

Despite the seven-month gap between experimental phases, memory trials in green turtles indicate that they have the ability to remember learned behaviour over a 9-month period (Drake et al., unpublished), and therefore, phase B changed the shape of the balloon in the hope of it being different enough to overcome the familiarity of phase A while retaining turtle interest and curiosity. This study assumes that the shape of the coloured object does not influence the response behaviour of the turtles. Balloons were fixed to a depth of 20 cm, which is half of the depth of the tank. None of the turtles appeared to have any trouble reaching and interacting with the balloons during the two phases. During phase A, both green and hawksbill turtles spent the majority of their time actively swimming at the surface but were seen diving during feeding. During phase B, hawksbill turtles spent the majority of time sitting on the floor of the tank, while greens still actively swam at the surface.

A significant behavioural shift was seen during the non-experimental period between phase A and phase B in which hawksbill turtles spent more time sitting on the bottom of the compartments and less time actively swimming. Collectively, hawksbill turtles were more likely to contact colours in phase A compared to phase B, and the amount of interaction with colours during phase B decreased greatly (Figure 1).

For the hawksbill turtles, the shape change may not have been different enough to overcome the memory of phase A and could also explain the slow contact time and overall decrease in interaction with the balloons. The behavioural differences seen between phase A and phase B in which hawksbills spent less time actively swimming and more time resting on the bottom of the compartment could suggest hawksbill turtles reaching ontogenetic shifts in diet or habitat settlement at some point between phase A and B, but further study into hawksbill development would be needed to test this hypothesis.

Green turtles had the opposite reaction between phase A and B to what the hawksbill turtles exhibited, where green turtles had a higher interaction with balloons and were faster to contact balloons in phase B than in phase A. Given there was no positive reward association for contacting coloured balloons, it is unclear why the green turtles had an increased response to the trials. There was no correlation between mass and latency time in either phase, suggesting green turtles’ increased interaction with the balloons was not a result of increased mass and likely not from any ontogenetic shifts in diet or habitat (Figure 2).

## 5. Conclusions

This study provides experimental support for species variation in reaction to colour between hawksbill and green turtles. Hawksbill turtles were most attracted to shorter wavelengths across a period of seven months from 15 to 22 months of age. Green turtles consistently preferred longer wavelengths across the same time period. This may reflect a different ecological need and associated visual capability and hue preference between these two species and age classes.

## Figures and Tables

**Table 1:** Behaviours in turtles during the white-coloured balloon control trials were determined and defined in an ethogram as per Table 1. This ethogram was applied to record behaviour in turtles during colour trials.

Behaviour	Explanation
Resting	For green turtles, front flippers were placed flat on the carapace and back flippers were either out to the sides or tucked under the plastron. Green turtles rested while floating on the surface of the water.
	For hawksbill turtles, front and back flippers were either positioned away from the body in a star shape or tucked under the plastron. Hawksbill turtles rested on the floor of the pen.
Diving	Turtle is completely submerged below the surface and resurfacing quickly.
Swimming (Wall Hugging)	Turtle swimming with plastron against the walls of the compartment, often lapping the full length of the compartment, or going from the wall opposite the balloon to the halfway area and then lapping back, remaining in the opposite half of the compartment from the balloon.
Swimming (Corner)	Turtle is facing away from balloon and actively swimming into a corner at the opposite end of the compartment from the balloon. Often, the turtle is pushing its head out of the water.
Splashing	Turtle slapping the surface of the water while quickly swimming around the compartment, usually while swimming along the walls or into a corner. Seen as a possible sign of agitation.
Balloon Touch (Flipper)	Turtle approaching a balloon close enough to hit with a front flipper. These touches were seen when a turtle was turning around to swim away from a balloon and not as a deliberate touch.
Balloon Touch (Carapace)	Turtle hitting a balloon with any part of the carapace, most often the back end, while turning around and swimming away from a balloon and therefore not regarded as a deliberate touch.
Carapace Turn	Turtle making a sharp turn in front of balloon exposing the majority of the carapace at a balloon. This behaviour was often repeated with turtle lapping the half of the compartment opposite to balloon and swimming quickly.
Balloon Touch (Beak)	Turtle touching a balloon with beak/mouth. Any form of beak to balloon contact was considered a deliberate touch including mouth open, mouth closed, from above or below the balloon.

## Data Availability

Data supporting results found in this study can be found at Research Data JCU-RDMP-Colour Vision in Turtles.
